# Tumor Suppressive Role of miR-342-5p and miR-491-5p in Human Osteosarcoma Cells

**DOI:** 10.3390/ph15030362

**Published:** 2022-03-16

**Authors:** Clément Veys, Manon Jammes, Françoise Rédini, Laurent Poulain, Christophe Denoyelle, Florence Legendre, Philippe Galera

**Affiliations:** 1 Normandie Univ., UNICAEN, BIOTARGEN, 14000 Caen, France; clement.veys@gmail.com (C.V.); manon.jammes@unicaen.fr (M.J.); florence.legendre@unicaen.fr (F.L.); 2 UMR 1238 Phy-Os “Bone Sarcomas and Remodeling of Calcified Tissues”, INSERM, Nantes University, 44035 Nantes, France; francoise.redini@univ-nantes.fr; 3 Normandie Univ., UNICAEN, INSERM U1086 ANTICIPE, Biology and Innovative Therapeutics for Ovarian Cancer (BioTICLA), 14000 Caen, France; l.poulain@baclesse.unicancer.fr (L.P.); c.denoyelle@baclesse.unicancer.fr (C.D.); 4 UNICANCER, Comprehensive Cancer Center F. Baclesse, 14000 Caen, France; 5 Normandie Univ., UNICAEN, Federative Structure Normandie Oncology, US Platon, ImpedanCELL Platform, 14000 Caen, France

**Keywords:** osteosarcoma, Bcl-2/Bcl-xL, miR-491-5p, miR-342-5p, Bak, apoptosis

## Abstract

Osteosarcomas are the most common type of malignant bone tumor. These tumors are characterized by the synthesis of an osteoid matrix. Current treatments are based on surgery and combination chemotherapy. However, for metastatic or recurrent tumors, chemotherapy is generally ineffective, and osteosarcomas are sometimes unresectable. Thus, the use of microRNAs (miRNAs) may represent an attractive alternative for the development of new therapies. Using high-throughput functional screening based on impedancemetry, we previously selected five miRNAs with potential chemosensitizing or antiproliferative effects on chondrosarcoma cells. We validated the tumor-suppressive activity of miR-491-5p and miR-342-5p in three chondrosarcoma cell lines. Here, we carried out individual functional validation of these five miRNAs in three osteosarcoma cell lines used as controls to evaluate their specificity of action on another type of bone sarcoma. The cytotoxic effects of miR-491-5p and miR-342-5p were also confirmed in osteosarcoma cells. Both miRNAs induced apoptosis. They increased Bcl-2 homologous antagonist killer (Bak) protein expression and directly targeted Bcl-2 lymphoma-extra large (Bcl-xL). MiR-342-5p also decreased B-cell lymphoma-2 (Bcl-2) protein expression, and miR-491-5p decreased that of Epidermal Growth Factor Receptor (EGFR). MiR-342-5p and miR-491-5p show tumor-suppressive activity in osteosarcomas. This study also confirms the potential of Bcl-xL as a therapeutic target in osteosarcomas.

## 1. Introduction

Osteosarcomas are the most common type of bone tumor [1,2,3]. They primarily affect children and adolescents. These tumors mainly develop in the metaphyseal region of long bones, such as the distal femur, the proximal tibia and the proximal humerus [4,5]. Osteosarcomas are bone-forming malignant tumors. They are classified into several histological subtypes: central, intramedullary and surface tumors, with subtypes in each group [5]. Conventional osteosarcomas represent 80% of all primary osteosarcomas. Conventional treatment of osteosarcomas consists of surgery in combination with chemotherapy, leading to an approximately 70% chance of patient remission after 5 years [6]. Nevertheless, the survival of patients with metastatic or recurrent tumors has remained unchanged over the past 40 years, with an overall survival rate of approximately 20% at 5 years [7]. At the time of diagnosis, 20% of patients have pulmonary metastases, but it is estimated that undetectable metastases are present in 80% of cases [8]. New therapeutic strategies are therefore needed for the treatment of osteosarcomas.

MicroRNAs (miRNAs) are short non-coding RNA transcripts of 18–24 nucleotides that regulate gene expression at the post-transcriptional or translational levels. They bind to the 3′-untranslated region (3′-UTR) of their target mRNA to suppress their translation or to stimulate their degradation [9]. Unlike short interfering RNAs (siRNAs), a single miRNA can silence the expression of multiple mRNAs, and the same RNA can be targeted by several miRNAs. Thus, miRNAs form complex regulatory networks in cell development, differentiation and homeostasis [10,11]. They have crucial functions in a wide variety of cellular processes, including proliferation, differentiation, apoptosis and metastasis. They can act either as oncogenes or tumor suppressors and may serve as diagnostic or prognostic biomarkers. Various studies have reported the aberrant expression of miRNAs in osteosarcomas [12,13,14]. Among them, miR-34a, a potent tumor suppressor, is downregulated in various human cancers, including osteosarcomas [15]. Qi-Cai Li et al. showed that miR-34a enhances sensitivity to cisplatin (CDDP) by upregulating the expression of c-Myc and Bcl-2-like protein 11 (Bim) and by inducing apoptosis in osteosarcoma cells [15]. MiRNAs may also enable the identification of novel therapeutic targets in osteosarcomas [13,14,15].

In a previous study, using functional high-throughput miRNA screening and in silico approaches, we identified five miRNAs that may stimulate apoptosis in a chondrosarcoma cell line by targeting Myeloid cell leukemia-1 mRNA (*MCL1*), Bcl-2 lymphoma-extra large mRNA (*BCL2L1*) or B-cell lymphoma-2 mRNA (*BCL2*) [16]. Individual functional studies were carried out with these miRNAs (miR-149-5p, miR-342-5p, miR-491-5p, miR-541-5p and miR-625-5p). We validated the apoptotic effects of miR-342-5p and miR-491-5p on three chondrosarcoma cell lines and identified the key signaling pathways involved in their tumor suppressor activity. Thus, we showed that miR-342-5p directly post-transcriptionally inhibits the anti-apoptotic *BCL2* and *BCL2L1* mRNAs and that miR-491-5p directly post-transcriptionally inhibits *BCL2L1* mRNA in SW1353 chondrosarcoma cells. Similar to a previous study [17], we also demonstrated that miR-491-5p inhibits the expression of Epidermal Growth Factor Receptor (EGFR), whose spontaneous activation in the absence of ligand is associated with tumor progression. Additionally, miR-491-5p also increases the expression of the Bak pro-apoptotic protein. Despite obvious pro-apoptotic effects, we also showed that miR-342-5p had anti-apoptotic potential because it leads to a decrease in pro-caspase-9 protein expression in chondrosarcoma cells [16].

In view of these results, here, we evaluated the effect of these five potential cytotoxic miRNAs on osteosarcoma cell lines to test their efficacy in other types of bone sarcoma cells and thereby determine the specificity of action of the miRNA effects. More specifically, we assessed the cytotoxic and chemosensitizing effects of miR-149-5p, miR-342-5p, miR-491-5p, miR-541-5p and miR-625-5p on three osteosarcoma cell lines. We observed cytotoxic effects for miR-342-5p and miR-491-5p only and, thereafter, performed individual functional validation studies with these two miRNAs. We validated their apoptotic effects and determined the key signaling pathways associated with their activity. In particular, using a luciferase reporter assay, we confirmed that miR-342-5p and miR-491-5p directly post-transcriptionally inhibit anti-apoptotic *BCL2L1* mRNA.

## 2. Results

### 2.1. MiR-342-5p and MiR-491-5p Have Antimetabolic Effects on Osteosarcoma Cell Lines

**Figure 1:**
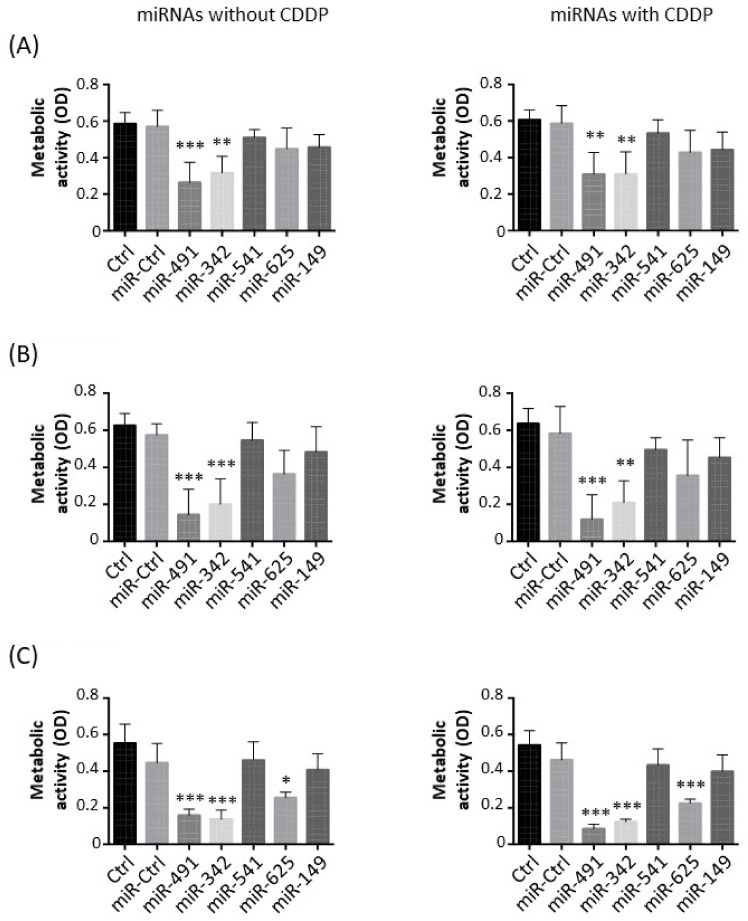
Effects of miRNAs on the metabolic activity of osteosarcoma cell lines. (**A**) HOS, (**B**) MG-63 and (**C**) SaOS-2 cells were grown for 24 h before transfection with 20 nM of the indicated miRNAs. After 48 h, cells were treated for 24 h with 0.5 µg/mL CDDP. The metabolic activity was measured 72 h after transfection. Data are presented as mean optical density (OD) ± SD of four independent experiments. Statistically significant differences between miR-Ctrl and miRNA-treated cells were determined using one-way ANOVA (***: *p* < 0.001, **: *p* < 0.01, *: *p* < 0.05).

We first evaluated the antimetabolic effects of miR-149-5p, miR-342-5p, miR-491-5p, miR-541-5p and miR-625-5p alone in the presence of a sublethal dose of cisplatin (CDDP) to test their chemosensitizing potential (Figure 1).

In the HOS cell line, miR-491-5p significantly decreased the metabolic activity compared with miR-Ctrl, both without CDDP (2.2-fold decrease, *p* < 0.001) and with CDDP (1.9-fold decrease, *p* < 0.01) (Figure 1A). MiR-342-5p also induced a significant decrease in the metabolic activity in both conditions (1.9-fold, *p* < 0.01). The other miRNAs did not significantly modulate the metabolic activity of HOS cells.

In the MG-63 cell line, only miR-491-5p and miR-342-5p significantly lowered the metabolic activity of the cells (Figure 1B). MiR-491-5p induced a 4-fold and 4.9-fold decrease (*p* < 0.001) in metabolic activity compared with miR-Ctrl and miR-Ctrl/CDDP-treated cells, respectively, with no significant difference between the two treatments. MiR-342-5p decreased metabolic activity by 2.9-fold in both conditions (*p* < 0.001 and *p* < 0.01 respectively).

Three miRNAs significantly impaired the metabolic activity of the SaOS-2 cell line (Figure 1C). MiR-625-5p decreased the metabolic activity by 1.7-fold (*p* < 0.05) relative to miR-Ctrl-treated cells and by 2-fold (*p* < 0.001) relative to miR-Ctrl/CDDP-treated cells. However, miR-491-5p and miR-342-5p had a more potent effect. MiR-491-5p downregulated the metabolic activity by 2.8-fold (*p* < 0.001) and by 5.4-fold (*p* < 0.001) compared with miR-Ctrl and miR-Ctrl/CDDP-treated cells, respectively. There was no significant difference in the metabolic activity of miR-491-5p- and miR-491-5p/CDDP-treated cells. Likewise, miR-342-5p decreased the metabolic activity by 3.2-fold without CDDP (*p* < 0.001) and by 3.5-fold with CDDP (*p* < 0.001).

Overall, these data demonstrate the antimetabolic effects of only miR-491-5p and miR-342-5p on all three osteosarcoma cell lines and of miR-625-5p only on the SaOS-2 cell line. Moreover, none of these miRNAs showed a significant chemosensitizing effect to CDDP on the metabolism of osteosarcoma cell lines.

### 2.2. MiR-342-5p and MiR-491-5p Have Cytotoxic Effects on Osteosarcoma Cell Lines

**Figure 2:**
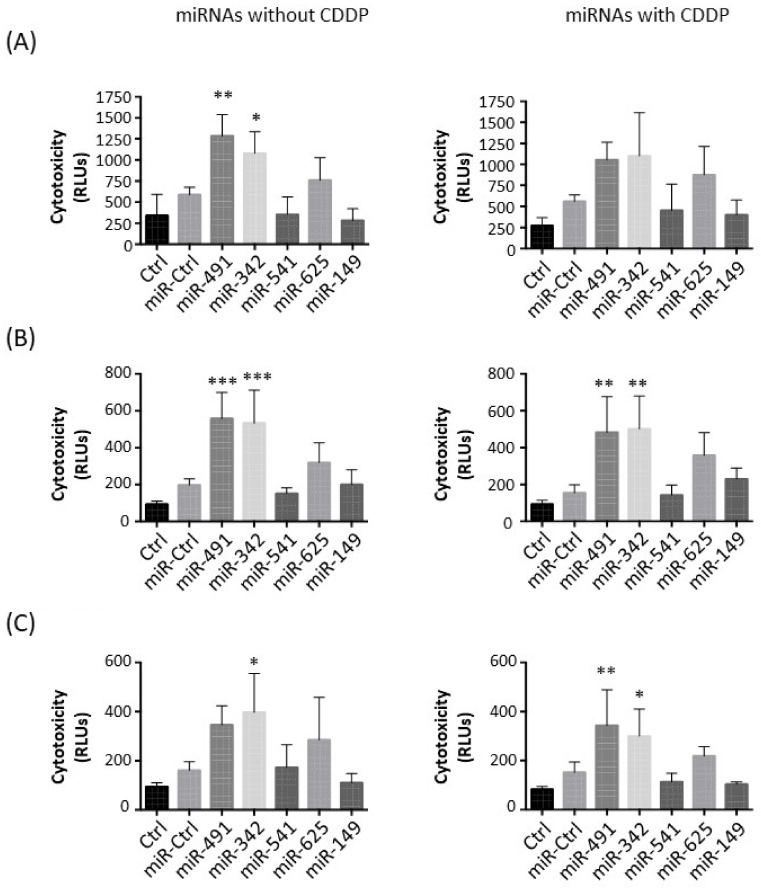
Evaluation of miRNA-induced cytotoxicity on osteosarcoma cell lines. (**A**) HOS, (**B**) MG-63 and (**C**) SaOS-2 cells were cultured and transfected with miRNAs as described in Figure 1. The cytotoxicity of miRNAs was measured 72 h after transfection. Data are presented as the mean relative luciferase units (RLU) ± SD of four independent experiments. Statistically significant differences between miR-Ctrl and miRNA-treated cells were determined using one-way ANOVA (***: *p* < 0.001, **: *p* < 0.01, *: *p* < 0.05).

Next, we studied the potential cytotoxic effect of these five miRNAs on the three osteosarcoma cell lines (Figure 2).

In the HOS cell line, miR-491-5p and miR-342-5p only induced significant cytotoxic effects when used alone: a 2.2-fold (*p* < 0.01) and a 1.8-fold increase (*p* < 0.05), respectively, compared with miR-Ctrl without CDDP (Figure 2A). In combination with CDDP, miR-491-5p and miR-342-5p did not significantly induce further cytotoxicity (1.9-fold increase and 2-fold increase, respectively). MiR-625-5p tended to increase cytotoxicity (1.3-fold increase relative to miR-Ctrl and 1.6-fold increase relative to miR-Ctrl/CDDP-treated cells).

Only miR-491-5p and miR-342-5p induced significant cytotoxic effects in MG-63 cells (approximately 3-fold increase with or without CDDP compared with miR-Ctrl) (Figure 2B). The effect of miR-625-5p was not statistically significant in the presence or absence of CDDP (1.6-fold and 2.3-fold increases, respectively).

In the SaOS-2 cell line, only miR-342-5p significantly triggered cytotoxic effects with and without CDDP (2.5-fold and 2-fold increases relative to the respective miR-Ctrl, *p* < 0.05), with no significant difference between the two conditions (Figure 2C). MiR-491-5p enhanced cytotoxicity by approximately 2-fold in both conditions, but this effect was only significant (*p* < 0.01) in combination with CDDP.

Only miR-491-5p and miR-342-5p showed significant cytotoxic effects on the three osteosarcoma cell lines. The combination of CDDP and miRNAs did not seem to improve the effects of miRNAs.

### 2.3. MiR-342-5p and MiR-491-5p Induce Cell Death in Osteosarcoma Cell Lines

**Figure 3:**
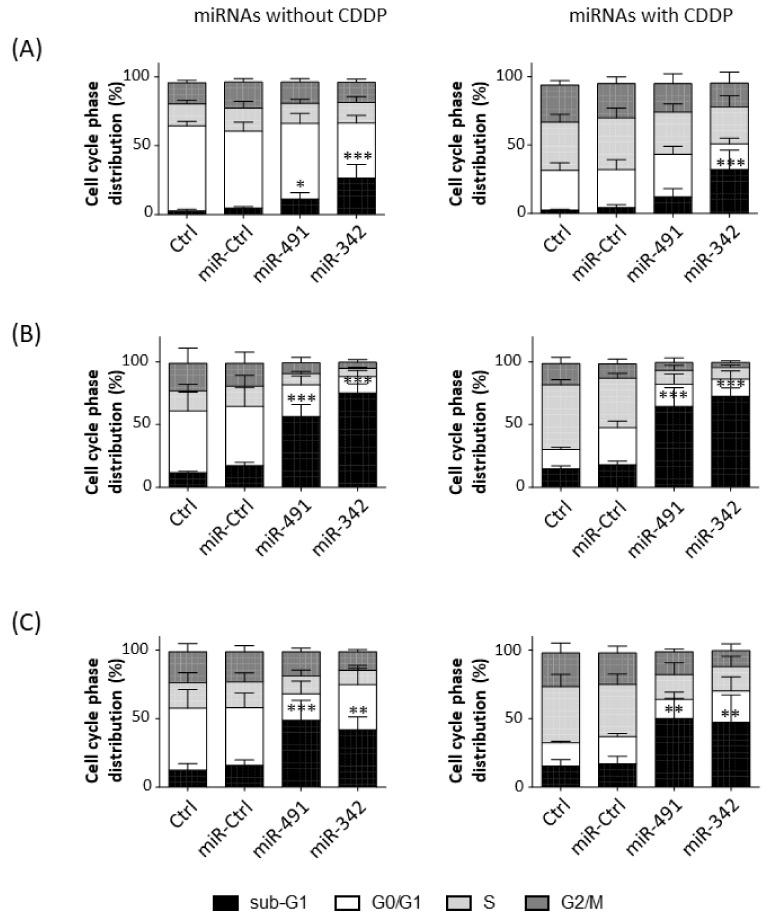
Evaluation of cytotoxic or chemosensitizing effects of miR-491-5p and miR-342-5p on osteosarcoma cell lines. (**A**) HOS, (**B**) MG-63 and (**C**) SaOS-2 cells were cultured and transfected with miR-Ctrl, miR-491-5p and miR-342-5p as described in Figure 1. Flow cytometry was carried out 72 h after transfection to analyze cell cycle phase distribution. The histograms show the analysis of five independent experiments for HOS cells and four independent experiments for MG-63 and SaOS-2 cells (mean ± SD with the different phases of the cycle). Statistically significant differences in the percentage of sub-G1 events between miR-Ctrl and miRNA-treated cells are presented. They were determined using one-way ANOVA (***: *p* < 0.001, **: *p* < 0.01, *: *p* < 0.05).

We then analyzed cell cycle progression in the three osteosarcoma cell lines in the presence or absence of a sublethal dose of CDDP (Figure 3).

CDDP increased the percentage of events in the S and G2/M phases, whereas it decreased the percentage of events in the G0/G1 phase in HOS cells (Figure 3A). CDDP enhanced the percentage of events only in the S phase in MG-63 and SaOS-2 cells (Figure 3B,C). MiR-491-5p and miR-342-5p alone did not induce a cell cycle blockade (no stalling in S or G2/M phases), but they increased the percentage of cells in the sub-G1 phase, an indicator of the induction of cell death.

In the HOS cell line, miR-491-5p increased the percentage of sub-G1 events with and without CDDP (by 2.4-fold and 2.9-fold relative to the respective miR-Ctrl (Figure 3A). This effect was significant only with miR-491-5p used alone (*p* < 0.05). MiR-342-5p induced a more significant increase in cell death, which increased by 5.6-fold and 7.5-fold with and without CDDP, respectively (*p* < 0.001). The proportions of cells in the sub-G1 phase were 4.8%, 11.3% and 26.7% without CCDP and 4.3%, 12.3% and 32.2% with CCDP for miR-Ctrl, miR-491-5p and miR-342-5p, respectively. Both miRNAs decreased cellular density 72 h post-transfection (Figure S1, Supplementary Materials). Moreover, we observed more cellular debris and condensed and/or fragmented chromatin, typical features of apoptotic cells (Figure S1).

In MG-63 cells, miR-491-5p and miR-342-5p induced a significant increase (*p* < 0.001) in the number of sub-G1 events (Figure 3B). For miR-491-5p, we observed a 3.2-fold increase without CDDP and a 3.6-fold increase with CDDP. MiR-342-5p increased cell death by 4.3-fold when used alone and by 4-fold in the presence of CDDP. The proportions of the cells in the sub-G1 phase were 17.4%, 56.4% and 75.3% without CCDP and 18%, 64.6% and 72.5% with CCDP for miR-Ctrl, miR-491-5p and miR-342-5p, respectively. A decrease in confluency and a high amount of cellular debris and apoptotic bodies were observed after the transfection of both miRNAs (Figure S2, Supplementary Materials).

In the SaOS-2 cell line, miR-491-5p enhanced the percentage of cells in the sub-G1 phase by 3-fold with and without CDDP (*p* < 0.001 and *p* < 0.01) compared with their respective miR-Ctrl (Figure 3C). MiR-342-5p also increased the number of sub-G1 events in both conditions (by 2.6-fold alone; by 2.8-fold with CDDP; *p* < 0.01). The proportions of cells in the sub-G1 phase were 16%, 48.8% and 41.8% without CCDP and 16.9%, 50% and 47.4% with CCDP for miR-Ctrl, miR-491-5p and miR-342-5p, respectively. This induction of cell death by both miRNAs was confirmed with microscopic observations at 72 h post-transfection (Figure S3, Supplementary Materials).

Overall, miR-342-5p and miR-491-5p functioned as apoptomiRs in HOS, MG-63 and SaOS-2 osteosarcoma cell lines. These miRNAs caused cell death at the same magnitude with and without CDDP, suggesting that they do not enhance the effect of CDDP.

### 2.4. MiR-342-5p and MiR-491-5p Activate the Apoptosis Pathway in Osteosarcoma Cells

**Figure 4:**
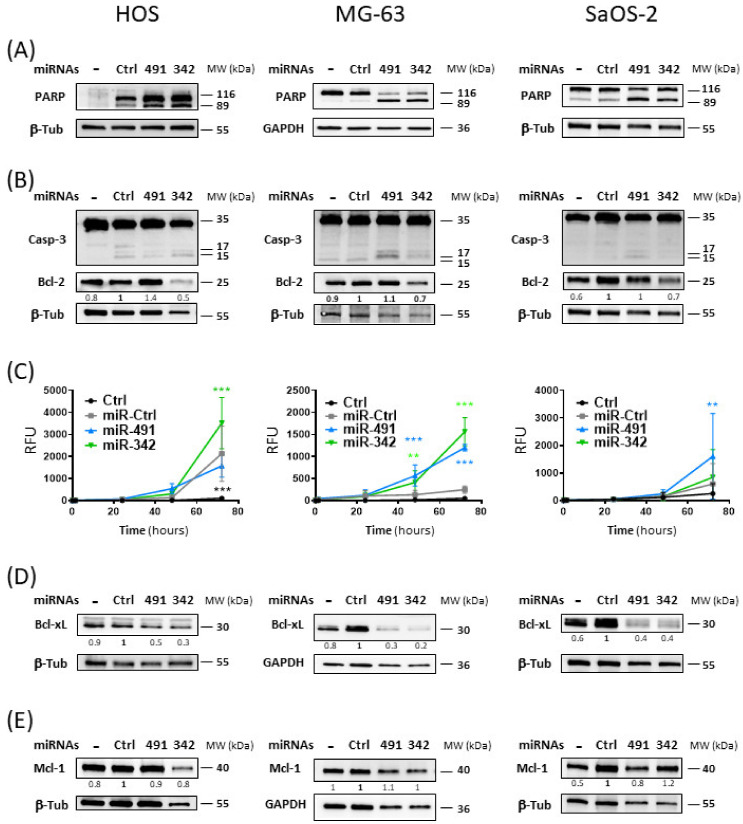
Apoptotic effects of miR-491-5p and miR-342-5p on osteosarcoma cell lines. In panels (**A**,**B**,**D**,**E**), HOS, MG-63 and SaOS-2 cells were grown for 24 h before transfection with 20 nM of miR-Ctrl, miR-491-5p and miR-342-5p for 72 h. Protein extracts were analyzed by Western blotting for (**A**) PARP, (**B**) caspase-3 and Bcl-2, (**D**) Bcl-xL and (**E**) Mcl-1 versus β-tubulin (β-Tub) or GAPDH. Representative blots of three independent Western blots are shown. Quantification of the bands of interest, normalized to β-tubulin or GAPDH signals and to miR-Ctrl, is shown below each blot. (**C**) Caspase-3/7 activity was measured in real time for 72 h after transfection as described in Section 4. The graphs show the analysis of fluorescence of four independent experiments performed in triplicate (mean relative fluorescent units (RFUs) ± SD). Statistically significant differences between miR-Ctrl and miRNA-treated cells were determined using two-way ANOVA (***: *p* < 0.001, **: *p* < 0.01).

We then investigated the induction of apoptosis by the two miRNAs used alone in the three osteosarcoma cell lines. MiR-491-5p and miR-342-5p led to poly(ADP-Ribose) Polymerase (PARP) cleavage in the three cell lines, with better cleavage detected with miR-491-5p compared with miR-342-5p in SaOS-2 cells (Figure 4A). In addition, both miRNAs also enhanced the cleavage of caspase-3 (Figure 4B) and induced caspase-3/7 activities (Figure 4C) to different extents, depending on the cell line.

In the HOS cell line, Western blotting showed that caspase-3 seemed to be more extensively cleaved with miR-342-5p than with miR-491-5p. Real-time analysis of caspase-3/7 activity revealed that only miR-342-5p significantly increased caspase-3/7 activity at 72 h post-transfection (by 1.4-fold, *p* < 0.001).

In the MG-63 cell line, we observed that the cleavage of caspase-3 was more pronounced with miR-491-5p than with miR-342-5p. Both miRNAs produced a significant increase in caspase-3/7 activity as early as 48 h post-transfection. At 72 h post-transfection, miR-342-5p enhanced caspase-3/7 activity by 6.2-fold (*p* < 0.001) and miR-491-5p by 4.8-fold (*p* < 0.001).

Cleavage of caspase-3 was only weakly detectable by Western blot with miR-491-5p in the SaOS-2 cell line. Similarly, only miR-491-5p significantly increased caspase-3/7 activity at 72 h post-transfection (by 2.7-fold, *p* < 0.01).

Because PARP cleavage by activated caspases and cleavage or activation of caspase-3/7 are characteristic of apoptosis, these results demonstrated that miR-491-5p and miR-342-5p induce apoptosis in osteosarcoma cells. MiR-342-5p appears to be the best apoptomiR in the HOS cell line, whereas miR-491-5p appears to be the best one in the SaOS-2 cell line. Both miRNAs were efficient in the MG-63 cell line, with miR-342-5p showing a better effect. These results are consistent with our previous results on cell death (Figure 3).

Both miRNAs potentially target members of the anti-apoptotic Bcl-2 family. Bcl-xL was validated as their direct target in the SW1353 chondrosarcoma cell line in our previous study [16]. Bcl-2 was also validated as a direct target of miR-342-5p in SW1353 cells. Mcl-1 was also a predicted target for both miRNAs [16]. We therefore studied their expression to evaluate their contribution to the induction of apoptosis by both miRNAs (Figure 4B,D,E).

Bcl-2 protein expression was decreased only upon miR-342-5p transfection (Figure 4B). The greatest inhibition was observed in the HOS cell line, with a 2-fold decrease compared with miR-Ctrl. MiR-342-5p downregulated Bcl-2 protein expression in the other two cell lines, but to a lesser extent (by 1.4-fold). We did not observe inhibition with miR-491-5p overexpression in any of the three cell lines, suggesting that Bcl-2 is not a target for this miR.

MiR-491-5p and miR-342-5p decreased Bcl-xL protein expression in the three osteosarcoma cell lines (Figure 4D). For miR-491-5p, we observed 2-fold, 3.3-fold and 2.5-fold decreases compared with miR-Ctrl in HOS, MG-63 and SaOS-2 cells, respectively. For miR-342-5p, we observed 3.3-fold, 5-fold and 2.5-fold decreases in HOS, MG-63 and SaOS-2 cells, respectively.

Although Mcl-1 was a predicted target of miR-491-5p and miR-342-5p, neither miRNA significantly modulated its protein expression in osteosarcoma cells, with a maximum decrease of 1.2-fold for miR-342-5p in HOS cells and for miR-491-5p in SaOS-2 cells (Figure 4E).

In summary, the inhibition of the expression of Bcl-xL and Bcl-2 may be responsible for the pro-apoptotic effect of miR-342-5p on osteosarcoma cells, and that of Bcl-xL may be involved in the pro-apoptotic effect of miR-491-5p.

### 2.5. MiR-342-5p and MiR-491-5p Target BCL2L1 mRNAs in the HOS Osteosarcoma Cell Line

We then investigated whether miR-342-5p and miR-491-5p directly bind the mRNAs of Bcl-2 (*BCL2* gene) and Bcl-xL (*BCL2L1* gene).

**Figure 5:**
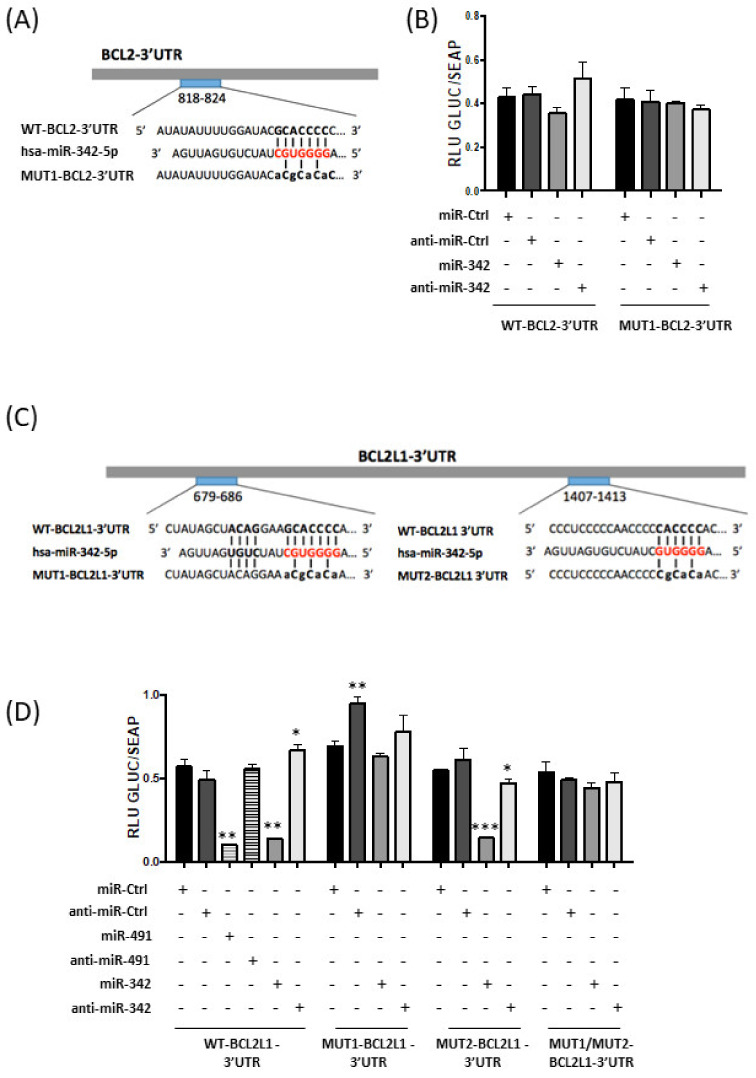
Analysis of inhibition of *BCL2* and *BCL2L1* mRNAs by miR-491-5p and miR-342-5p in HOS cells. (**A**) Schematic representation of wild-type (WT) and mutant (MUT1) BCL2-3′UTR with their predicted hsa-miR-342-5p binding sites. (**B**) Luciferase reporter constructs containing WT and MUT1 BCL2-3′UTR (1 ng/μL) were transfected into HOS cells concomitantly with the indicated miR or anti-miR (20 nM). (**C**) Schematic representation of wild-type (WT) and mutant (MUT1 and MUT2) BCL2L1-3′UTR with their predicted hsa-miR-342-5p binding sites. The MUT1/MUT2-BCL2L1-3′UTR vector contains both mutations 1 and 2. (**D**) Luciferase reporter constructs containing WT and mutant BCL2-3′UTR (2 ng/μL) were transfected into HOS cells concomitantly with the indicated miR or anti-miR (20 nM). (B, D) Secreted alkaline phosphatase (SEAP) and Gaussia luciferase (GLUC) assays were carried out 48 h post-transfection. GLUC activity was normalized to SEAP activity and expressed as relative luciferase units (RLU). Representative experiments (mean ± SD) of four independent experiments performed in triplicate are provided. Statistically significant differences between miR-Ctrl and miRNA- or anti-miRNA-treated cells were evaluated using a two-tailed Student’s *t*-test (***: *p* < 0.001, **: *p* < 0.01, *: *p* < 0.05).

A putative binding site for miR-342-5p is located between nucleotides 818 and 824 in BCL2-3′UTR (Figure 5A). Transfection of miR-342-5p reduced the luciferase activity of the WT-BCL2-3′UTR reporter vector by 17% compared with miR-Ctrl (Figure 5B). By contrast, co-transfection with anti-miR-342-5p increased luciferase reporter activity (by 20%). A mutation in the BCL2-3′UTR binding site prevented the inhibitory effect of miR-342-5p. Nevertheless, the inhibitory effect of miR-342-5p on BCL2-3′UTR was not statistically significant. Therefore, we cannot unambiguously confirm that *BCL2* mRNA is a direct target of miR-342-5p in HOS cells.

Computational analysis predicted two potential binding sites for miR-342-5p in BCL2L1-3′UTR at positions 679–686 and 1407–1413. Several constructs were generated with two individual mutations in these sites (MUT1 and MUT2, respectively) and a construct with both mutations (MUT1/MUT2) (Figure 5C).

Only co-transfection of miR-342-5p and WT-BCL2L1-3′UTR or MUT2-BCL2L1-3′UTR resulted in strong repression of luciferase activity (−76% and −74%, *p* < 0.01 and *p* < 0.001, respectively; Figure 5D). By contrast, miR-342-5p did not significantly reduce the reporter activity for the MUT1-BCL2L1-3′UTR construct or for the double-mutant MUT1/MUT2-BCL2L1-3′UTR construct. Additionally, co-transfection of anti-miR-342-5p led to an increase in luciferase activity for the WT-BCL2L1-3′UTR construct (+17%, *p* < 0.05) and only decreased the luciferase activity by 14% (*p* < 0.05) for the MUT2-BCL2L1-3′UTR construct. In conclusion, in HOS cells, miR-342-5p represses Bcl-xL protein expression through direct and preferential binding to BCL2L1-3′UTR at positions 679–686.

We co-transfected miR-491-5p with WT-BCL2L1-3′UTR bearing three binding sites for miR-491-5p in HOS cells (Figure 5D). This co-transfection substantially decreased the luciferase activity (−82% compared to miR-Ctrl, *p* < 0.01). This repression was not obtained with anti-miR-491-5p. These results demonstrate that miR-491-5p also directly targets the 3′UTR of *BCL2L1* mRNA in the HOS osteosarcoma cell line.

### 2.6. MiR-342-5p and MiR-491-5p Regulate the Expression of Numerous Proteins Related to Apoptosis or Proliferation

Next, we studied the expression of some of their validated or predicted targets involved in cell survival or cell death.

**Figure 6:**
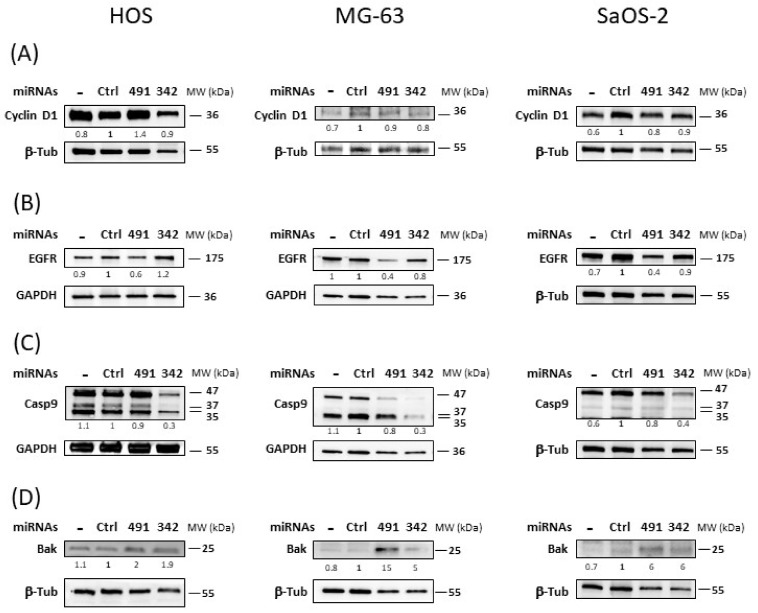
Analysis of some survival/death proteins affected by miR-491-5p and miR-342-5p in osteosarcoma cell lines. HOS, MG-63 and SaOS-2 were cultured and transfected for 72 h as described in Figure 4. Protein extracts were analyzed by Western blotting for (**A**) cyclin D1, (**B**) EGFR, (**C**) caspase-9 and (**D**) Bak versus β-tubulin (β-Tub) or GAPDH. Representative blots of three independent Western blots are shown. Quantification of the bands, normalized to β-tubulin or GAPDH signals and to miR-Ctrl, is shown below each blot.

Transfection of miR-342-5p did not significantly downregulate the expression of the cyclin D1 protein in any of the three osteosarcoma cell lines (Figure 6A). The inhibition did not exceed 1.2-fold in MG-63 cells. We did not observe the inhibition of cyclin D1 protein expression by miR-491-5p in the three osteosarcoma cell lines.

MiR-491-5p inhibited EGFR protein expression by 1.6-fold in HOS cells and by 2.5-fold in MG-63 and SaOS-2 cells (Figure 6B). By contrast, miR-342-5p had no significant inhibitory effect on EGFR in any of the three osteosarcoma cell lines.

In the three osteosarcoma cell lines, miR-342-5p induced a strong decrease in pro-caspase-9 protein expression (47 kDa; by 3.3-fold in HOS and MG-63 cells and by 2.5-fold in SaOS-2 cells) (Figure 6C). This decrease was not due to the induction of large cleaved caspase-9 fragments (35 and 37 kDa). MiR-491-5p did not modulate pro-caspase-9 protein expression nor induce protein cleavage.

The pro-apoptotic protein Bak was positively regulated by both miRNAs in all three osteosarcoma cell lines (Figure 6D). For miR-491-5p, we observed a 2-fold, 15-fold and 6-fold increase compared with miR-Ctrl in HOS, MG-63 and SaOS-2 cells, respectively. For miR-342-5p, we detected a 1.9-fold, 5-fold and 6-fold upregulation in HOS, MG-63 and SaOS-2 cells, respectively.

In summary, miR-491-5p inhibited the expression of EGFR, a protein involved in cell survival, and it enhanced the expression of Bak, a pro-apoptotic protein in osteosarcoma cells. MiR-342-5p also increased Bak protein expression, but at the same time, it decreased pro-caspase-9 protein expression, a caspase involved in the apoptosis pathway.

## 3. Discussion

Osteosarcomas represent the most frequent forms of bone sarcomas, with an annual prevalence of 1 in 100,000. Today, osteosarcomas are generally well-treated tumors due to the effectiveness of combination chemotherapy associated with surgery. However, for patients with metastases or recurrence, the survival rate is much lower. As a consequence, treatment seems to have a low success rate, which has not improved for many years. It is therefore essential to develop new tools for the diagnosis and therapy for this type of tumor.

The use of therapeutic miRNAs offers the perspective of a multi-target approach and can help uncover new therapeutic targets for clinical applications [13,14,15]. Using miRNA screening based on real-time cell analysis, we found five miRNAs (miR-149-5p, miR-342-5p, miR-491-5p, miR-541-5p and miR-625-5p) capable of decreasing cell proliferation in the SW1353 chondrosarcoma cell line [16]. We validated the cytotoxic and apoptotic effects of miR-342-5p and miR-491-5p but not of miR-149-5p, miR-541-5p and miR-625-5p on 3 chondrosarcoma cell lines. Four of these miRNAs (all but miR-541-5p) have already been studied in other types of cancer. MiR-149-5p and miR-625-5p were able to suppress cancer migration and invasion and reverse drug resistance [18,19,20]. We therefore tested these five miRNAs on three osteosarcoma cell lines.

For miR-541-5p, miR-625-5p and miR-149-5p, we did not observe any significant cytotoxic effect after 72 h incubation. However, for miR-625-5p, we detected a slight increase in the cytotoxic effect on the three cell lines, as well as significant downregulation in the metabolic activity of the SaOS-2 cell line. It is possible that a longer incubation period is required to observe cell death effects or chemosensitizing effects for these miRNAs. Indeed, no significant effect of miR-149-5p overexpression was observed on SaOS-2 and U-2OS proliferation after 3 days of incubation in a MTT assay, whereas antiproliferative effect was observed at 4 days [21]. Chemosensitization of miR-625-5p to CDDP was observed in an MTT assay after 48 h of incubation with CDDP in two multidrug-resistant gastric cancer cell lines, whereas we performed the XTT assay after 24 h in osteosarcoma cell lines [18]. In our previous study performed on chondrosarcoma cell lines [16], only miR-342-5p and miR-491-5p clearly showed significant antimetabolic and cytotoxic effects, as in the present study on osteosarcoma. We then studied the effects of miR-342-5p and miR-491-5p on the cell cycle. Both miRNAs enhanced the number of sub-G1 events, reflected by the presence of cellular debris, a hallmark of apoptosis, in the three cell lines. MiR-342-5p was the most effective inducer of cell death in the HOS cell line. In MG-63 cells, both miRNAs induced an equivalent increase in the number of sub-G1 events, with a slightly better effect for miR-342-5p, and vice versa in SaOS-2 cells.

Even though we did not previously observe chemosensitizing effects of miR-491-5p and miR-342-5p in chondrosarcoma cells, we evaluated the ability of these miRNAs to enhance the sensitivity of the three osteosarcoma cell lines to CDDP. Neither of the two miRNAs was able to increase sensitivity to CDDP. However, we chose to use a sublethal dose of CDDP (1.65 µM) to only induce cell cycle arrest in the S and/or G2/M phases for the three cell lines, rather than doses close to the inhibitory concentrations to reach 50% reduction in cell viability (IC_50_), as has been used by others. In the literature, IC_50_ values for the three cell lines are very variable, depending on the incubation time and the viability assays used (from 3.5 µM to 78 µM for HOS cells, 1.65 to 26 µM for MG-63 cells and 4.9 µM to 46 µM for SaOS-2 cells) [22,23,24]. MG-63 seemed to be the least resistant. We checked whether these concentrations of CDDP induced more cell death to validate the relative sensitivity of the cell lines to a CDDP treatment alone (Figure S4, Supplementary Materials). Indeed, elevated concentrations of CDDP increased the percentage of cells in the sub-G1 phase rather than cause a cell cycle blockade in the S and G2-M phases for the HOS cell line and in the S phase for the MG-63 and SaOS-2 cell lines. We hypothesized that cell drug resistance was too high with sublethal doses of CDDP to see a chemosensitizing effect of miRNAs. Moreover, after the transfection of miRNA, we incubated the cells only for 24 h with CDDP versus 48 h in most of the previous studies, as already mentioned for miR-625-5p [18]. Another study demonstrated that the overexpression of miR-491-5p was able to sensitize osteosarcoma cell lines (MG-63 and U2OS) after incubation with 10 µM CDDP for 48 h [25]. Therefore, the chosen kinetics and the sublethal dose of CDDP may not be adequate to reveal any chemosensitizing effect of miRNAs, as in our previous study [16]. Other experiments are required to explore this hypothesis.

**Figure 7:**
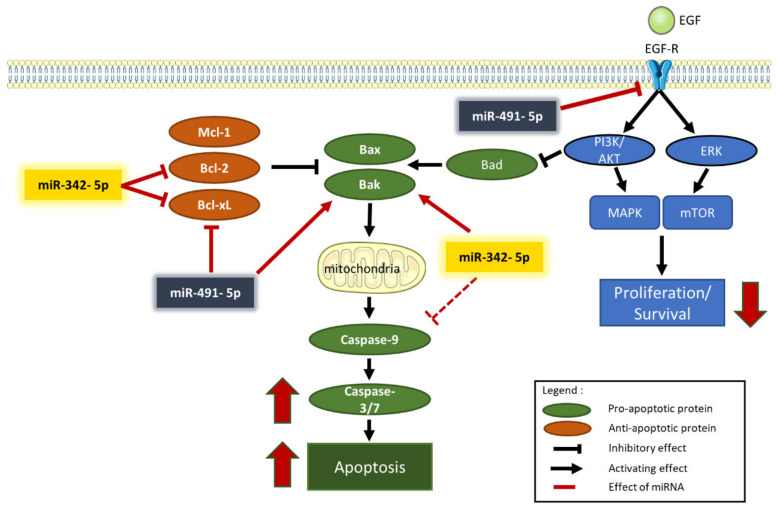
Overview of the mechanisms of action of miR-491-5p and miR-342-5p in human osteosarcoma cells.

The analysis of the expression of proteins such as PARP and caspase-3 allowed us to confirm that apoptosis is involved in miRNA-induced cell death. MiR-342-5p and miR-491-5p induced PARP cleavage in the three osteosarcoma cell lines. We also revealed an enhancement of caspase-3/7 activity, although to different extents, with both miRNAs (Figure 7). Because miR-Ctrl also increased caspase-3/7 activity in HOS and SaOS-2 cells, the results appeared to be less significant than in MG-63 cells. We also observed PARP cleavage for the miR-Ctrl condition in Western blots with HOS and SaOS-2 cells. However, we clearly demonstrated that miR-342-5p is the best apoptosis inducer in the HOS cell line, whereas miR-491-5p is the best inducer in the SaOS-2 cell line. Both miRNAs significantly induced caspase-3/7 activity in the MG-63 cell line, with miR-342-5p showing a stronger effect. These results correlate with the data obtained using flow cytometry.

MiR-491-5p has previously been studied in osteosarcoma. One study showed that this miRNA is downregulated in osteosarcoma tissues and cell lines [26]. Another study demonstrated that the level of miR-491-5p decreases in the serum of osteosarcoma patients and correlates with increased metastasis, chemoresistance and thus a lower survival rate [25]. The overexpression of miR-491-5p has been shown to restore the chemosensitivity of osteosarcoma cells to CDDP and to directly target *CRYAB* encoding crystallin alpha B, a protein that inhibits apoptosis and contributes to intracellular architecture [25]. The latter study showed that miR-491-5p exerts its tumor-suppressing activity by directly targeting *CRYAB*. Other direct targets of miR-491-5p have been identified in osteosarcoma cells, such as *FOXP4* and *PKM2* (encoding forkhead-box P4 and pyruvate kinase muscle 2, respectively) [26,27]. Their inhibition increases apoptosis or suppresses cell proliferation of osteosarcoma cells. Nevertheless, the mechanisms involved in the expression of miR-491-5p and its biological role in osteosarcomas still need to be explored.

In cancer, constitutive activation of EGFR is associated with cell proliferation, survival and migration. Expression of EGFR has frequently been observed in high-grade osteosarcomas. EGFR does not appear to be a major driver for osteosarcoma cell growth, but it may contribute to starvation and chemotherapy resistance [28]. *EGFR* mRNA is a validated target of miR-491-5p in other types of cancers, such as glioblastomas and ovarian carcinomas [17,29]. As previously shown in chondrosarcoma cell lines [16], miR-491-5p decreased the level of EGFR expression in osteosarcoma cell lines, which may contribute to a decrease in the cell proliferation rate, as shown in Figure 7.

Several studies have shown that overexpression of miR-491-5p directly inhibits the expression of the Bcl-xL protein in various cancers [17,30,31]. We also previously demonstrated this direct inhibition in SW1353 chondrosarcoma cells [16]. In the present study, we confirmed that miR-491-5p induced cell death in the HOS cell line by directly targeting *BCL2L1* mRNA and consequently decreasing Bcl-xL protein expression. By contrast, although the anti-apoptotic member Mcl-1 may be a putative target of miR-491-5p, this miRNA did not influence Mcl-1 expression in any of the three osteosarcoma cell lines investigated. On the other hand, overexpression of miR-491-5p increased the expression level of the pro-apoptotic protein Bak. Inhibition of Bcl-xL expression and overexpression of Bak may therefore contribute to its apoptotic activity in osteosarcoma cells (Figure 7), as in chondrosarcoma cells [16].

Only one study has investigated the effect of miR-342-5p in osteosarcomas. It directly targeted the Wnt Family member 7b (*Wnt7b*) oncogene, inhibited tumor growth, migration and invasion and restored cell sensitivity to doxorubicin [32]. However, in other studies, including ours, miR-342-5p has been shown to be a tumor suppressor in neuroblastomas, colon cancers, ovarian cancer cells and chondrosarcomas [16,33,34,35]. Although *EGFR* and *MCL1* mRNA may be potential targets of miR-342-5p, our data did not show significant inhibition of their protein expression in the three osteosarcoma cell lines analyzed. *CCND1* mRNA is a validated target of miR-342-5p in neuroblastomas [33], but as in chondrosarcoma cell lines [16], we did not observe downregulated cyclin D1 expression in any of the three osteosarcoma cell lines.

MiR-342-5p decreased Bcl-2 protein expression (from 1.4 to 2-fold) in the three osteosarcoma cell lines. Although we validated *BCL2* mRNA as a direct target of miR-342-5p in our previous study on chondrosarcomas [16], here, we did not validate *BCL2* mRNA as a real direct target of miR-342-5p. Transfection of miR-342-5p decreased the luciferase activity of the WT-BCL2-3′UTR vector in HOS cells by 1.2-fold, but it was not statistically significant. We suspect that the pro-apoptotic effects of miR-342-5p are linked to Bcl-xL inhibition rather than to Bcl-2 inhibition in osteosarcoma cells. In effect, miR-342-5p downregulated Bcl-xL protein expression from 2.5 to 5-fold in osteosarcoma cell lines and significantly decreased the activity of the WT-BCL2L1-3′UTR vector by 4.2-fold in HOS cells. As shown for neuroblastomas [33], we validated *BCL2L1* mRNA as a direct target of miR-342-5p. Moreover, as in our previous study on chondrosarcomas [16], we also clearly identified its binding site at position 679-686 of BCL2L1-3′UTR. Overexpression of Bak also contributes to the apoptotic effects of miR-342-5p (Figure 7).

We showed that miR-342-5p also had anti-apoptotic effects on the three osteosarcoma cell lines by reducing pro-caspase-9 protein expression (by approximately 3-fold). We did not observe the induction of caspase-9 cleavage by either miRNA, but, unlike other caspases, caspase-9 showed complete activity in its uncleaved form [36]. These results are consistent with our previous study on chondrosarcoma cells [16] and with another study on cardiomyocytes [36]. In osteosarcoma cells, miR-342-5p clearly induced apoptosis by inducing caspase-3/7 activity and PARP cleavage but could also have some anti-apoptotic potential (Figure 7).

In osteosarcomas, overexpression of Bcl-2 and Bcl-xL has been reported to be associated with poor prognosis [37,38]. Metastatic or recurrent osteosarcomas have a high expression of Bcl-xL, which is correlated with a low probability of survival, mainly due to resistance to chemotherapy [38]. This correlation between Bcl-xL expression and chemoresistance in cancers is very common and has been demonstrated in a panel of 60 cell lines [39]. Additionally, a study showed that pharmacological inhibition of Bcl-xL improves the sensitivity of osteosarcomas to doxorubicin [40]. Here, miR-491-5p and miR-342-5p targeted *BCL2L1* mRNA and induced apoptosis. We thus confirmed the potential of Bcl-xL as a relevant therapeutic target in osteosarcomas.

## 4. Material and Methods

### 4.1. Cell Culture

The osteosarcoma cell lines HOS, MG-63 and SaOS-2 were kindly provided by Dr. F. Rédini (INSERM UMR 1238 Phy-Os, Nantes, France). The osteosarcoma cell line HOS (ATCC^®^ CRL-1543^TM^) was grown in high-glucose Dulbecco’s modified Eagle’s medium (HG-DMEM, L0103-500, Biowest, Nuaillé, France) supplemented with 10% fetal calf serum (FCS) (CVFSVF00-01, Eurobio Scientific, Courtaboeuf, France). In addition, antibiotics and an antifungal were added to the media (100 U/mL penicillin, 100 µg/mL streptomycin and 0.25 µg/mL amphotericin B (CABPSA00-0U, Eurobio Scientific, Courtaboeuf, France)).

The osteosarcoma cell line MG-63 (ATCC^®^ CRL-1427^TM^) was cultured in Eagle’s minimum essential medium (MEM, CM1MEM18-01, Biowest, Nuaillé, France) supplemented with 10% FCS (CVFSVF00-01, Eurobio Scientific, Courtaboeuf, France). A cocktail of 100 U/mL penicillin and 100 µg/mL streptomycin (CABPE501-0U, Eurobio Scientific, Courtaboeuf, France) was added to the medium.

The osteosarcoma cell line SaOS-2 (ATCC^®^ HTB-85^TM^) was grown in DMEM/Nutrient Mixture F-12 Ham (DMEM:HAM’s F12, CM1MHA60-01, Biowest, Nuaillé, France) supplemented with 5% FCS (CVFSVF00-01, Eurobio Scientific, Courtaboeuf, France) and 2 mM L-glutamine (CSTGLU00-0U, Eurobio Scientific, Courtaboeuf, France). A cocktail of 100 U/mL penicillin and 100 µg/mL streptomycin (CABPE501-0U, Eurobio Scientific, Courtaboeuf, France) was added to the medium. The absence of mycoplasma was confirmed by PCR. All cells were incubated at 37 °C in a 5% CO_2_ atmosphere.

### 4.2. Drugs and miRNAs

MiRNA mimics and miRNA hairpin inhibitors were purchased from Dharmacon (Horizon Discovery, Cambridge, UK). MiR-Control (MIMAT0000039), hsa-miR-149-5p (MIMAT0000450), hsa-miR-342-5p (MIMAT0004694), hsa-miR-491-5p (MIMAT0002807), hsa-miR-541-5p (MIMAT0004919) and hsa-miR-625-5p (MIMAT0003294) were purchased from Dharmacon (Horizon Discovery, Cambridge, UK) and used as previously described [16]. Hsa-miR-342-5p-hairpin inhibitor (IH-301083-02), hsa-miR-491-5p-hairpin inhibitor (IH-300751-06) and miRNA hairpin inhibitor negative control (IN-001005-01) were also purchased from Dharmacon and used as previously described [16]. Cisplatin (CDDP) was purchased from Mylan (3019071509, Merck, Santé SAS, Lyon, France). The same sublethal dose of CDDP (0.5 µg/mL or 1.65 µM) was used during the transfection of miRNA for all osteosarcoma cell lines.

### 4.3. Transfection of miRNA and CDDP Treatment

Exponentially growing HOS, MG-63 and SaOS-2 cells were seeded at 1 × 10^4^ cells/cm^2^. Twenty-four hours after seeding, cells were transfected with 20 nM miRNA using INTERFERin^TM^ (409-10, Polyplus-Transfection, Strasbourg, France) following the manufacturer’s protocol. After 48 h, cells were treated with a sublethal dose of CDDP.

### 4.4. Metabolic Activity Analysis

HOS, MG-63 and SaOS-2 cells were seeded onto 96-well microplates at a density of 3.2 × 10^3^ cells/well in triplicate. Cells were grown for 24 h before transfection of 20 nM miRNA. Treatment with CDDP was initiated 48 h later, as described above. Cell metabolic activity was assessed 72 h after transfection. XTT assay (11465015001, Roche, Basel, Switzerland) was performed as previously described [16]. Absorbance was measured after 1 h of incubation with a microplate reader (Spark 10M, Tecan Lyon, France) at 450 nm with a reference wavelength at 600 nm.

### 4.5. Cytotoxicity Assay

Cells were cultured under the same conditions as for the XTT assays. Seventy-two hours after transfection of miRNA, cytotoxicity was evaluated with a bioluminescence cytotoxicity assay kit (CA4680, Interchim, Montluçon, France). Adenylate kinase detection was carried out in the supernatant as previously described [16]. Luminescence was measured with a microplate reader (Spark 10M, Tecan Lyon, France).

### 4.6. Cell Cycle Analysis

Adherent cells were harvested by trypsinization. Detached cells and trypsinized cells were pooled, rinsed and fixed in 70% ethanol as previously described [16]. Fixed cells were treated with propidium iodide (20 µg/mL, P4170-10MG, Sigma Aldrich, Saint-Louis, MO, USA) and RNase (100 µg/mL, 12091-021, Fisher Scientifics SAS, Illkirch, France) as previously described [16]. DNA content was analyzed using the Cytoflex S Flow Cytometer (Beckman Coulter France SAS, Paris, France). Data were analyzed by Cytexpert^®^ acquisition software (Beckman Coulter France SAS, Paris, France).

### 4.7. Analysis of Nuclear Morphology

Morphological characterization of apoptotic cells by nuclear staining with DAPI (sc-24941, Santa Cruz Biotechnology, Dallas, TX, USA) was performed after cyto-centrifugation as previously described [16]. Cell confluence and nuclear staining were monitored using a fluorescence microscope (Nikon Eclipse Ti; Nikon Instruments Inc., Melville, NY, USA).

### 4.8. Western Blotting

Total proteins were extracted from both detached and adherent cells using ice-cold RIPA lysis buffer as previously described [41]. Protein concentrations were determined using the Bradford assay (500-0006, BioRad, Hercules, CA, USA). Protein levels were analyzed by immunoblotting with antibodies from Cell Signaling Technology (Ozyme, Saint-Quentin-en-Yvelines, France): caspase-9 (9502), cyclin D1 (2878), Bak (3814), Bcl-xL (2764), EGFR (4267) and PARP (9542), as previously described [16]. GAPDH (FL-335), Mcl-1 (S-19) and β-tubulin (D-10) were from Santa Cruz Biotechnology (Dallas, TX, USA). Bcl-2 (M0887) was from DAKO (Agilent, Santa Clara, CA, USA). Appropriate horseradish-conjugated secondary antibodies (115-035-003 and 111-035-003, Jackson Immunoresearch, West Grove, PA, USA) were used as previously described [16]. Proteins were detected using enhanced chemiluminescence (170-5061, BioRad Hercules, CA, USA), and signals were visualized with the ChemiDoc MP Imaging System (BioRad, Hercules, CA, USA). Signals were quantified using ImageLab^®^ software (Biorad, Hercules, CA, USA).

### 4.9. Luciferase miRNA Target Reporter Assay

Wild-type (WT) and mutant (MUT) vectors of BCL2-3′UTR (217HmiT016211a-MT05) and BCL2L1-3′UTR (217HmiT108616-MT05) containing Targetscan predictive or mutated miR-342-5p and miR-491-5p binding sites were purchased from GeneCopoeia (Rockville, MD, USA) and used as previously described [16]. Briefly, they contain a Gaussia luciferase (GLUC) reporter gene and a secreted alkaline phosphatase (SEAP) reporter gene upstream 3′UTR sequences. HOS cells were seeded at 4 × 10^4^ cells/well (triplicates) in a 24-well plate. After 24 h, they were transfected with miRNA mimic or miRNA hairpin inhibitor (20 nM each) and with BCL2-3′UTR vectors (1 ng/µL) using jetOPTIMUS^TM^ DNA transfection reagent (117-01, Polyplus-Transfection, Strasbourg, France). Endofectin transfection reagent (EF013, GeneCopoeia, Rockville, MD, USA) was used to transfect BCL2L1-3′UTR vectors (2 ng/µL) and miRNAs. Secreted alkaline phosphatase (SEAP) and Gaussia luciferase (GLUC) activities were assessed 48 h after transfection in the culture medium with the secrete-pair dual luminescence assay kit (LF032, GeneCopoeia, Rockville, MD, USA). SEAP and GLUC activities were measured with a microplate reader (Spark 10M; Tecan, Lyon, France).

### 4.10. Real-Time Detection of Caspase-3/7-Mediated Apoptosis

Caspase-3/7 activity was assessed using the Incucyte^®^ Caspase-3/7 Green Apoptosis Assay Reagent (C10723, Fisher Scientifics SAS, Illkirch, France) as previously described [16]. Briefly, HOS, MG-63 and SaOS-2 cells seeded at 3.2 × 10^3^ cells/well were grown in 96-well plates. They were monitored after transfection of 20 nM miRNA for 72 h in the Incucyte^®^ S3 by acquiring images (objective × 10; Essen BioScience, Ltd., Royston, United Kingdom) every 3 h in two separate regions per well. Experiments were analyzed with the Incucyte^®^ software (Essen BioScience, Ltd., Royston, United Kingdom).

### 4.11. Statistical Analysis

Experiments were independently repeated at least three times. Values are reported as means ± SD. Statistical analyses were performed using one-way ANOVA corrected for multiple comparisons using Dunnett’s test or two-way ANOVA corrected for multiple comparisons using Sidak’s test. Alternatively, statistical significance was assessed by two-tailed unpaired Student’s *t*-test with Welch’s correction to analyze statistical differences within representative experiments performed in triplicate. Data were analyzed with GraphPad Prism 7 software (San Diego, CA, USA): *** *p* < 0.001, ** *p* < 0.01 and * *p* < 0.05.

## 5. Conclusions

We clearly demonstrated the antimetabolic, cytotoxic and antiproliferative effects of miR-342-5p and miR-491-5p on three osteosarcoma cell lines and also their pro-apoptotic effects. Unfortunately, they failed to induce chemosensitivity to CDDP in our experimental conditions, as observed in previous impedancemetry experiments. We also showed that miR-342-5p and miR-491-5p directly target *BCL2L1* mRNA. Moreover, miR-491-5p inhibited the protein expression of EGFR and increased that of Bak. MiR-342-5p also increased the protein expression of Bak and decreased that of Bcl-2 and Bcl-xL but, at the same time, decreased pro-caspase-9 expression. Overall, this study highlighted the functional and essential role of Bcl-xL in osteosarcoma cells and confirmed the therapeutic potential to target Bcl-xL in osteosarcomas.

## Data Availability

Data are contained within the article and Supplementary Materials.

## Supplementary Materials

The following supporting information can be downloaded at: https://www.mdpi.com/article/10.3390/ph15030362/s1, Figure S1: Characterization of the effects of miR-491-5p and miR-342-5p on HOS cells; Figure S2: Characterization of the effects of miR-491-5p and miR-342-5p on MG-63 cells; Figure S3: Characterization of the effects of miR-491-5p and miR-342-5p on SaOS-2 cells. Figure S4: CDDP dose–response in osteosarcoma cell lines.
