# Supplementary material for: Tumor Suppressive Role of miR-342-5p and miR-491-5p in Human Osteosarcoma Cells
Source: Pharmaceuticals (Basel). 2022 Mar 16;15(3):362. doi: 10.3390/ph15030362 (PMC8949568; doi:10.3390/ph15030362)
Supplement: Supplementary file 1 [file pharmaceuticals-15-00362-s001.zip › pharmaceuticals-1524972-supplementary.pdf]

pharmaceuticals

Article

Tumor Suppressive Role of miR-342-5p and miR-491-5p in Human Osteosarcoma Cells

Clément Veys [1], Manon Jammes [1], Françoise Rédini [2], Laurent Poulain [3,4], Christophe Denoyelle [3,4,5], Florence Legendre [1,†] and Philippe Galera [1,*,†]

[1] Normandie Univ., UNICAEN, BIOTARGEN, 14000 Caen, France; clement.veys@gmail.com (C.V.); manon.jammes@unicaen.fr (M.J.); florence.legendre@unicaen.fr (F.L.)
[2] UMR 1238 Phy-Os "Bone Sarcomas and Remodeling of Calcified Tissues", INSERM, Nantes University, F-44035 Nantes, France; francoise.redini@univ-nantes.fr (F.R.)
[3] Normandie Univ., UNICAEN, INSERM U1086 ANTICIPE, Biology and Innovative Therapeutics for Ovarian Cancer (BioTICLA), 14000 Caen, France; l.poulain@baclesse.unicancer.fr (L.P.); c.denoyelle@baclesse.unicancer.fr (C.D.)
[4] UNICANCER, Comprehensive Cancer Center F. Baclesse, 14000 Caen, France
[5] Normandie Univ., UNICAEN, Federative Structure Normandie Oncology, US Platon, ImpedanCELL Platform, 14000 Caen, France
* Correspondence: philippe.galera@unicaen.fr
† These authors contribute equally to the work.

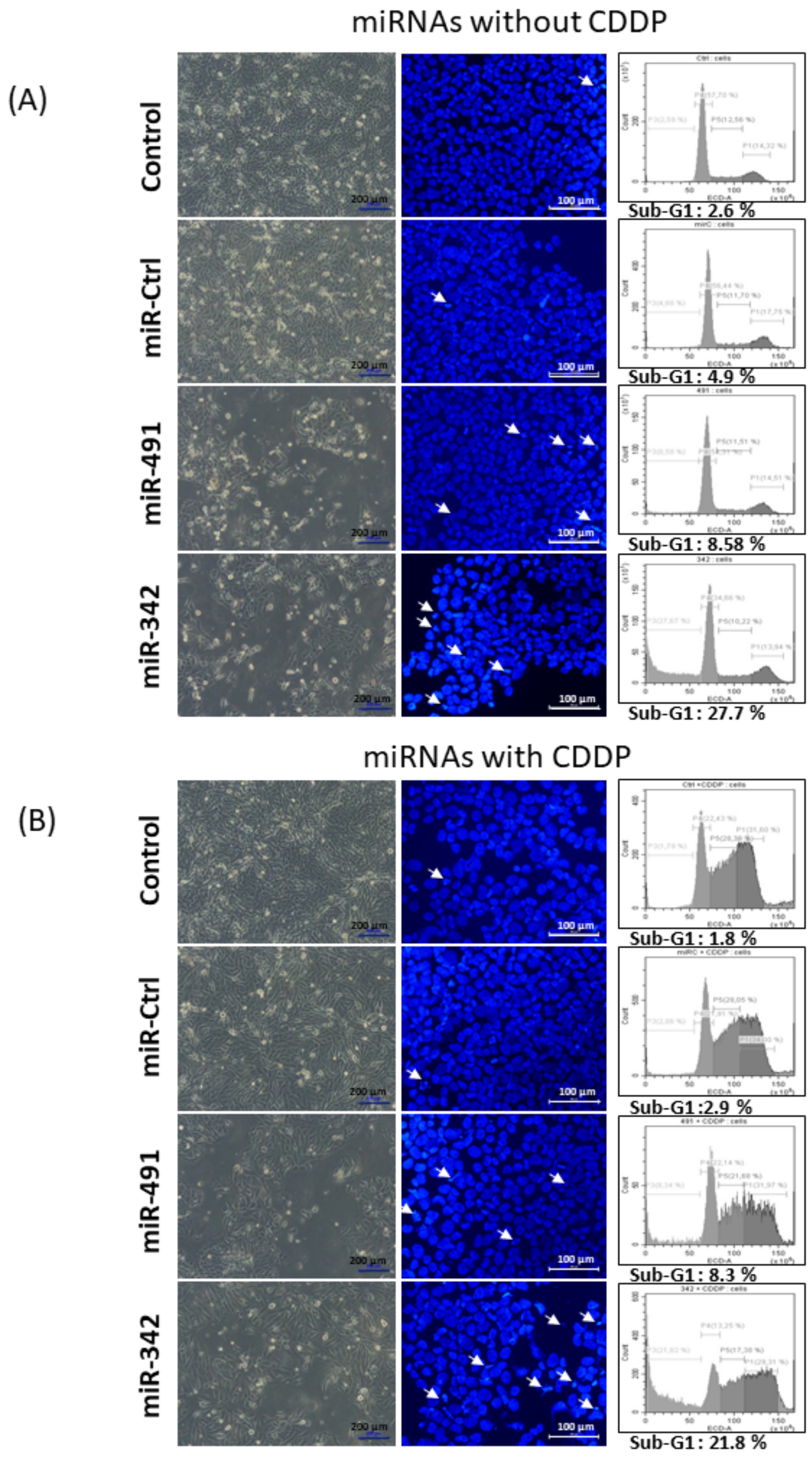
miRNAs without CDDP
(A)
Control
Sub-G1 : 2.6 %
miR-Ctrl
Sub-G1 : 4.9 %
miR-491
Sub-G1 : 8.58 %
miR-342
Sub-G1 : 27.7 %
200 µm
100 µm
miRNAs with CDDP
(B)
Control
Sub-G1 : 1.8 %
miR-Ctrl
Sub-G1 :2.9 %
miR-491
Sub-G1 : 8.3 %
miR-342
Sub-G1 : 21.8 %

Figure S1. Characterization of the effects of miR-491-5p and miR-342-5p on HOS cells. HOS cells were transfected with miRNAs 24 h after seeding as described in Figure 3. Treatment with cisplatin (CDDP) (0.5 µg/ml) was performed 48 h post-transfection for 24 h. Analyses were carried out 72 h post-transfection. In the left part of each panel, cell morphology was obtained with photonic microscopy at the end of the experiment. In the middle part of each panel, nuclear morphology was obtained after DAPI staining. White arrowheads show cells with condensed and/or fragmented chromatin and cellular debris. In the right part of each panel, DNA content histograms were obtained using flow cytometry. Images shown are representative of five independent experiments.

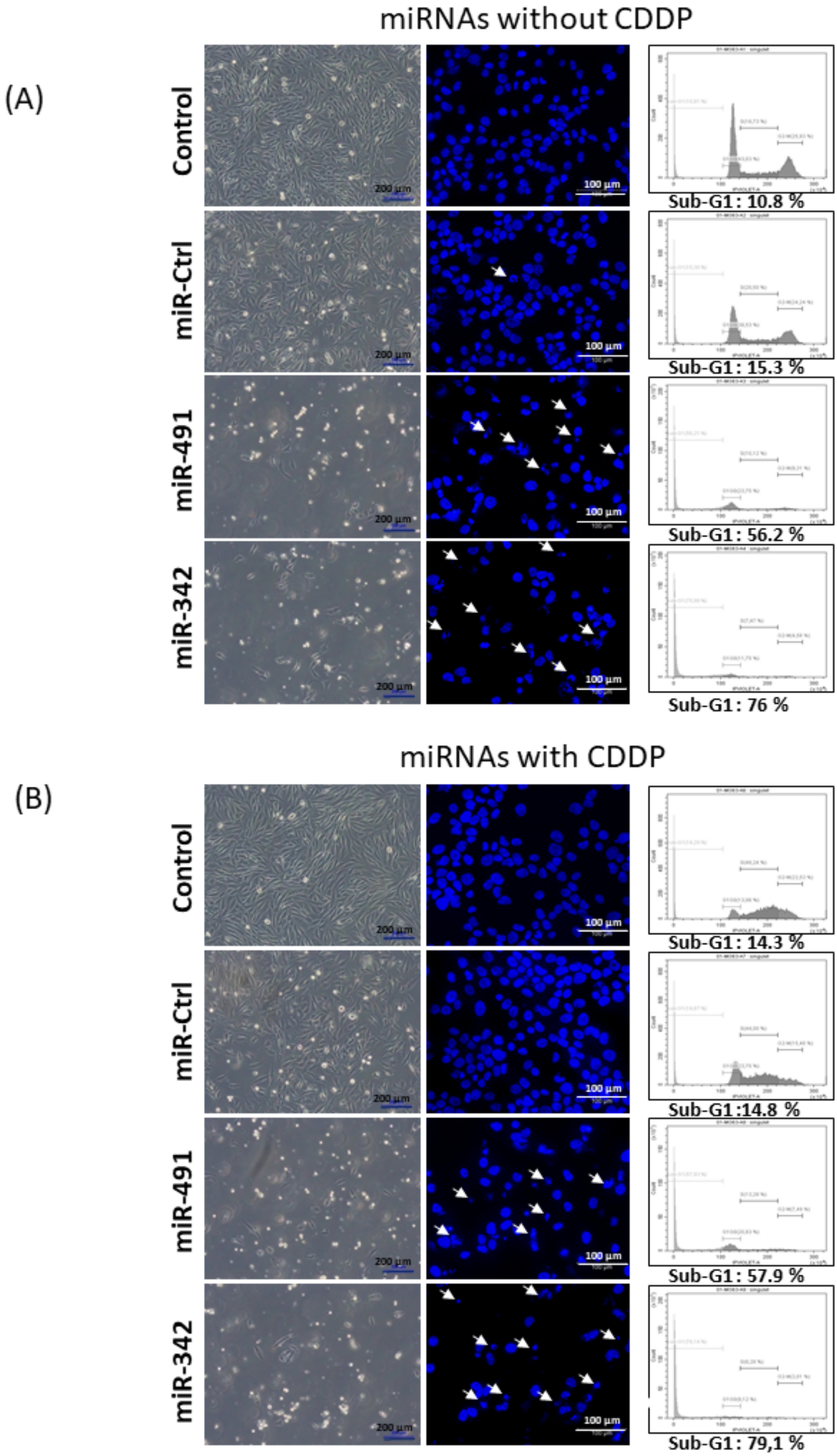
miRNAs without CDDP
(A)
Control
miR-Ctrl
miR-491
miR-342
200 µm
100 µm
Sub-G1 : 10.8 %
Sub-G1 : 15.3 %
Sub-G1 : 56.2 %
Sub-G1 : 76 %
miRNAs with CDDP
(B)
Control
miR-Ctrl
miR-491
miR-342
Sub-G1 : 14.3 %
Sub-G1 :14.8 %
Sub-G1 : 57.9 %
Sub-G1 : 79,1 %

Figure S2. Characterization of the effects of miR-491-5p and miR-342-5p on MG-63 cells. MG-63 cells were transfected with miRNAs 24 h after seeding as described in Figure 3. Treatment with cisplatin (CDDP) (0.5 µg/ml) was performed 48 h post-transfection for 24 h. Analyses were carried out 72 h post-transfection. In the left part of each panel, cell morphology was obtained with photonic microscopy at the end of the experiment. In the middle part of each panel, nuclear morphology was obtained after DAPI staining. White arrowheads show cells with condensed and/or fragmented chromatin and cellular debris. In the right part of each panel, DNA content histograms were obtained using flow cytometry. Images shown are representative of four independent experiments.

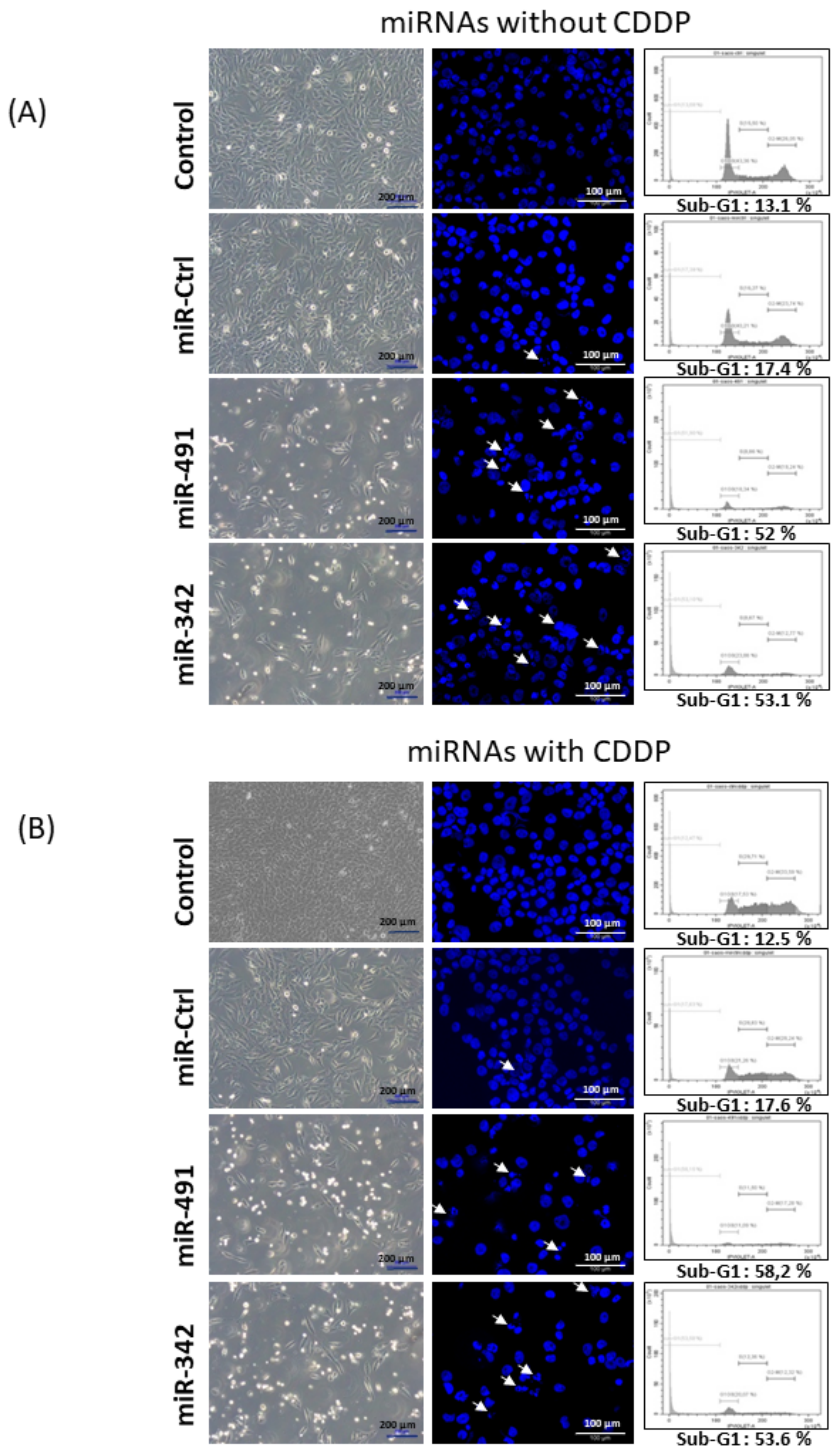
miRNAs without CDDP
(A)
Control
miR-Ctrl
miR-491
miR-342
200 µm
100 µm
Sub-G1 : 13.1 %
Sub-G1 : 17.4 %
Sub-G1 : 52 %
Sub-G1 : 53.1 %
miRNAs with CDDP
(B)
Control
miR-Ctrl
miR-491
miR-342
Sub-G1 : 12.5 %
Sub-G1 : 17.6 %
Sub-G1 : 58,2 %
Sub-G1 : 53.6 %

Figure S3. Characterization of the effects of miR-491-5p and miR-342-5p on SaOS-2 cells. SaOS-2 cells were transfected with miRNAs 24 h after seeding as described in Figure 3. Treatment with cisplatin (CDDP) (0.5 µg/ml) was performed 48 h post-transfection for 24 h. Analyses were carried out 72 h post-transfection. In the left part of each panel, cell morphology was obtained with photonic microscopy at the end of the experiment. In the middle part of each panel, nuclear morphology was obtained after DAPI staining. White arrowheads show cells with condensed and/or fragmented chromatin and cellular debris. In the right part of each panel, DNA content histograms were obtained using flow cytometry. Images shown are representative of four independent experiments.

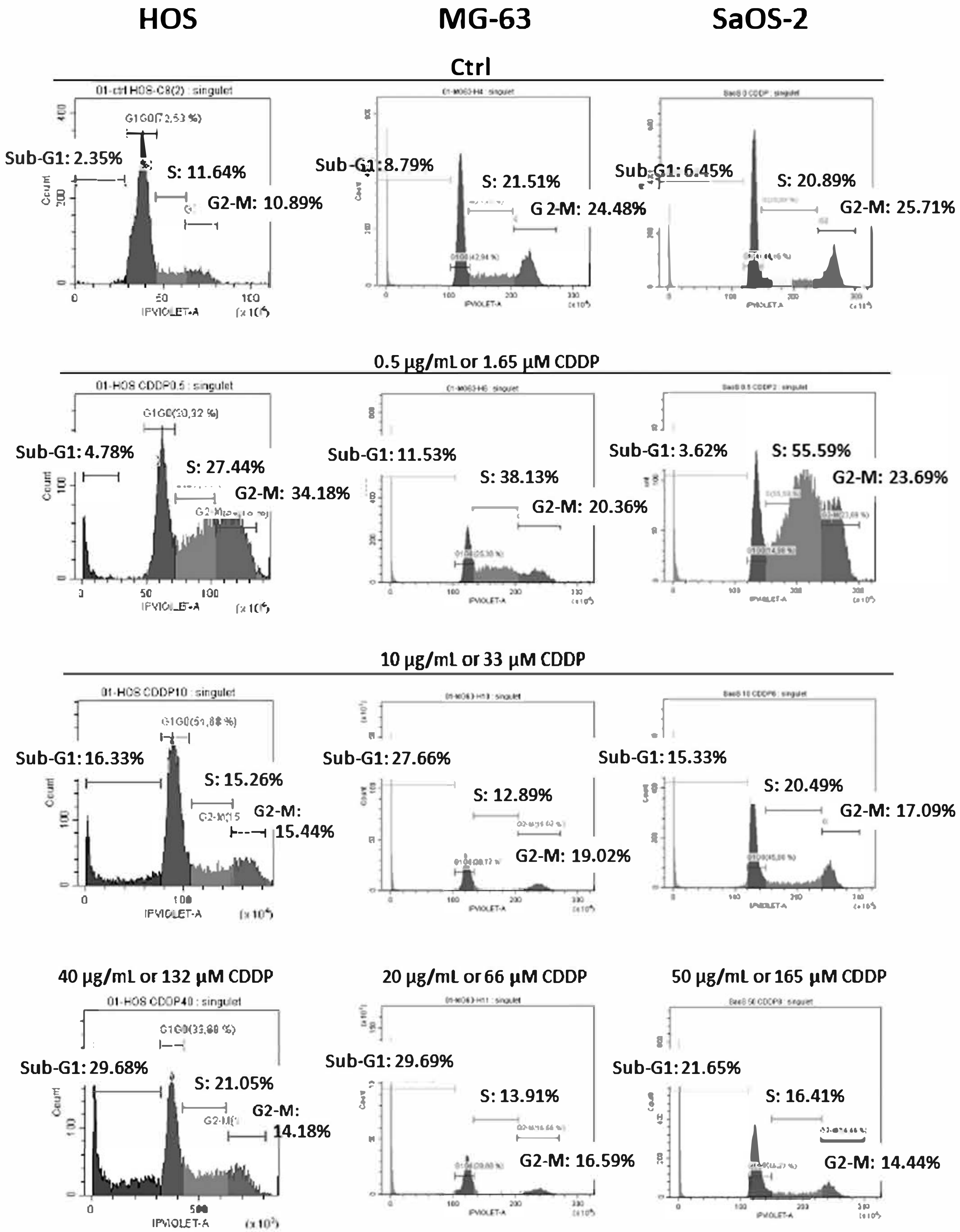
HOS
MG-63
SaOS-2
Ctrl
Sub-G1: 2.35%
S: 11.64%
G2-M: 10.89%
Sub-G1: 8.79%
S: 21.51%
G 2-M: 24.48%
Sub-G1: 6.45%
S: 20.89%
G2-M: 25.71%
0.5 µg/mL or 1.65 µM CDDP
Sub-G1: 4.78%
S: 27.44%
G2-M: 34.18%
Sub-G1: 11.53%
S: 38.13%
G2-M: 20.36%
Sub-G1: 3.62%
S: 55.59%
G2-M: 23.69%
10 µg/mL or 33 µM CDDP
Sub-G1: 16.33%
S: 15.26%
G2-M: 15.44%
Sub-G1: 27.66%
S: 12.89%
G2-M: 19.02%
Sub-G1: 15.33%
S: 20.49%
G2-M: 17.09%
40 µg/mL or 132 µM CDDP
20 µg/mL or 66 µM CDDP
50 µg/mL or 165 µM CDDP
Sub-G1: 29.68%
S: 21.05%
G2-M: 14.18%
Sub-G1: 29.69%
S: 13.91%
G2-M: 16.59%
Sub-G1: 21.65%
S: 16.41%
G2-M: 14.44%

Figure S4. CDDP dose-response in osteosarcoma cell lines.

HOS, MG-63 and SaoS-2 cells were treated 72 h after seeding with cisplatin (CDDP) for 24 h at the indicated concentrations (0.5, 10, or 20, or 40 or 50 μg/mL). DNA content histograms were obtained using flow cytometry. Images shown are representative of two independent experiments.
